# Experimental demonstration of broadband light trapping by exciting surface modes of an all-dielectric taper

**DOI:** 10.1038/s41598-019-39906-8

**Published:** 2019-03-05

**Authors:** Tsung-Yu Huang, Ta-Jen Yen

**Affiliations:** 1 Department of Materials Science and Engineering, National Tsing Hua University, Hsinchu, 30013 Taiwan Republic of China; 2 Department of Materials Engineering, Ming Chi University of Technology, New Taipei City, 24301 Taiwan Republic of China; 3 Center for Nanotechnology, Materials Science, and Microsystems, National Tsing Hua University, Hsinchu, 30013 Taiwan Republic of China

## Abstract

We design an all-dielectric taper and then excite its surface modes by illuminating a plane wave upon the taper to achieve broadband light trapping spanning from 20 to 100 GHz. Via Lewin’s theory, such excitation of surface modes could be analogous to “trapped rainbow”, i.e., activation of negative Goos-Hänchen effect within a negative refractive waveguide. To further reinforce this statement, the corresponding power flow distributions within the all-dielectric taper are recorded in finite-integration time domain simulation and suggest that a chromatic incident pulse is indeed trapped at different critical thicknesses of the taper, a character of the negative refractive waveguide. Finally, the transmittance is measured and compared to the simulated ones. The two followed the similar trend.

## Introduction

Slow light has been attracting many researchers’ attention due to its ability to manipulate photons and regarded as a possible candidate to transmit information for innovative features of all optical computation and communication. Till now, there are various systems to slow and even to stop light, such as electromagnetic induced transparency (EIT) for quantum interferences^1,2^, plasmonic analogue EIT for coupled bright and dark atoms^3,4^, photonic crystals for negative Goos-Hänchen effect^5,6^ and negative refractive waveguides (NRWs) for both surface plasmon polariton (SPP)^7,8^ and oscillatory modes^9–11^. Nevertheless, there appeared several associated challenges, for example, non-solid-state devices for quantum EIT^1^, narrow bandwidth for plasmonic analogue EIT and photonic crystals^6,9^, and large sensitivity to roughness of interfaces for SPP modes of NRWs^10^. These challenges have impeded practical applications of these aforementioned systems. In contrast, oscillatory modes of NRWs distribute their field maxima, instead of at the interfaces like SPP modes, within the bodies of the waveguides, thus preventing its sensitivity to interfaces’ roughness; on the other hand, simply employing a broadband negative index material as a core of the waveguide to support oscillatory modes enables a clear observation of the so-called “trapped rainbow”^11–13^, i.e., broadband light trapping compared to other slow light systems. The broadband light trapping system play a crucial role in the fields of energy harvesting and optical computation and could be applied to enhancing energy conversion efficiency of solar cells^14–16^ and realizing quantum optical memory, storage and data processor^11,17^. Therefore, it is oscillatory modes of NRWs, the most promising candidate to slow or even to stop light. Nevertheless, although several broadband negative index metamaterials (NIM), for example, silver-dendritic left-handed metamaterials^18^ and crescent meta-atoms from transformation optics^19^ have been proposed, the establishment of a broadband NIM seems to mainly rely on usage of metallic structures that would inevitably introduce intrinsic Ohmic loss into the systems to degrade or even destroy negative refraction of the core. Such constraints of realizing a broadband NIM have hindered practical applications of a slow light system. Therefore, in this work, we would like to design an all-dielectric taper that supports Mie resonance-based surface modes. These surface modes could be analogue to ‘trapped rainbow’ in a tapered NRW once illuminated by a plane wave. More importantly, the employment of the all-dielectric taper could not only render the system free from intrinsic Ohmic loss but also prolongs interaction time between matters and photons.

To discuss an all-dielectric taper, we would briefly elucidate surface modes of a small particle, including metallic and dielectric particles. Derived in 1908 by Gustav Mie^20^, surface modes of a small particle feature a novel and unprecedented absorption ability, which is unseen for the bulk of the same material. A famous example of surface modes is the one excited from a metallic particle and usually known as localized surface plasmons (LSPs) with so many different applications such as biosensors^21^ and enhanced second harmonic generation^22^. On the contrary, surface modes of a small dielectric particle, also denominated as ‘material polaritons’^23^, have received little attention compared to LSPs; yet, material polaritons not only adopt the characteristic of LSPs (e.g., morphology-dependent resonances and their sensitivity to environments) but also further eliminate intrinsic Ohmic loss from conventional LSPs. Thus, it is possible to achieve broadband light trapping via an all-dielectric taper as compared to the metallic one^18,24,25^; herein, in lieu of exciting LSPs in a metallic particle, we intend to excite material polaritons in the all-dielectric taper to trap incident waves. Noteworthily, we can easily determine resonance frequencies of surface modes of the taper from apexes of electric and magnetic scattering coefficients (*b*_*n*_ & *a*_*n*_, respectively) since the excitation conditions of surface modes are equivalent to that the denominators of the scattering coefficients equal to 0^26,27^.

## Results

### Design of an all-dielectric taper

**Figure 1:**
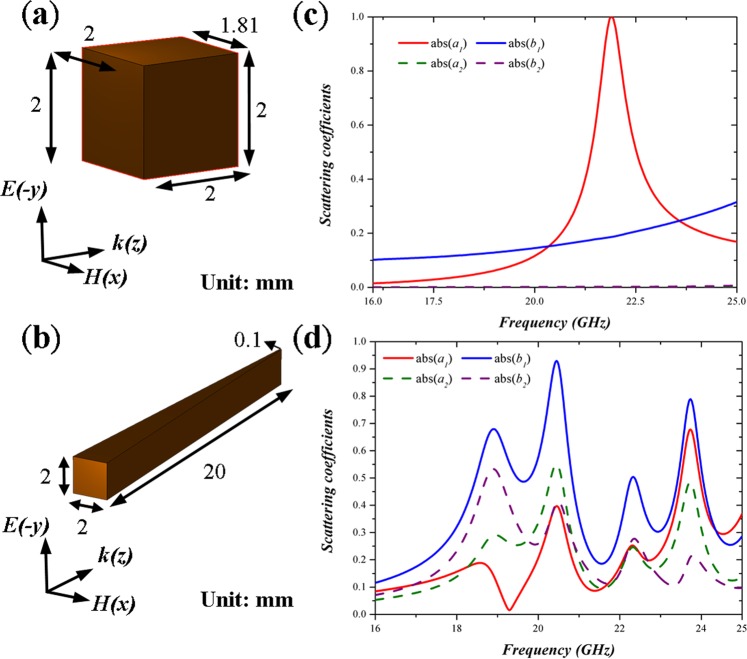
Detailed dimension and excitation conditions of (**a**) a shorter all-dielectric taper and (**b**) a longer all-dielectric taper. Corresponding scattering coefficients of (**c**) the shorter and (**d**) longer dielectric taper. According to these peaks, we could identify possible resonance frequencies of surface modes within the tapers and such surface modes enable broadband light trapping.

To design an all-dielectric taper, we choose zirconia, ZrO_2_ as the building material due to its relatively large dielectric constant (*ε*_*r*_ = 33) and small loss tangent (*tan δ*  = 0.02) at microwave region. In this section, we would like to discuss two different cases, a shorter dielectric taper and a longer dielectric taper. The shorter dielectric taper is with a width of 2 mm in the front end and 1.81 mm in the back end, a constant height of 2 mm through the taper and a length of 2 mm as shown in Fig. 1(a). On the other hand, the longer dielectric taper possesses a width of 2 mm in the front end and 0.1 mm in the back end, a constant height of 2 mm, and a length of 20 mm as shown in Fig. 1(b). Here, we can regard the longer dielectric taper as a stack of many different shorter dielectric tapers with different varied sizes. The tapers are excited by plane waves with y-polarization. Under such excitation condition, the longer taper structure is expected to support surface modes at a wider frequency range based on morphology-dependent Mie resonances to achieve broadband light trapping. Figure 1(c,d) illustrates absolute values of electric and magnetic scattering coefficients (*a*_1_, *b*_1_, *a*_2_ and *b*_2_) of the shorter and longer dielectric tapers, respectively. The scattering coefficients are derived from the orthogonality of spherical elementary waves (see Method for detailed discussion). We can clearly observe a single magnetic resonance peak around 21.9 GHz for the shorter taper; in contrast, if we consider the longer one, multi-resonance peaks are observed (i.e., 18.58 and 20.45 GHz for magnetic resonances and 18.91 and 20.45 GHz for electric resonances), thus providing an evidence for the longer taper to support excitation of so many surface modes to enable broadband light trapping. After confirming the resonance frequencies of the surface modes of the longer all-dielectric taper, we employ finite-integration-time-domain (FITD) simulation method to retrieve its complex reflectance and transmittance, then deriving absorbance based on

1 $${\rm{A}}({\rm{\omega }})=1-{\rm{R}}({\rm{\omega }})-{\rm{T}}({\rm{\omega }})$$

**Figure 2:**
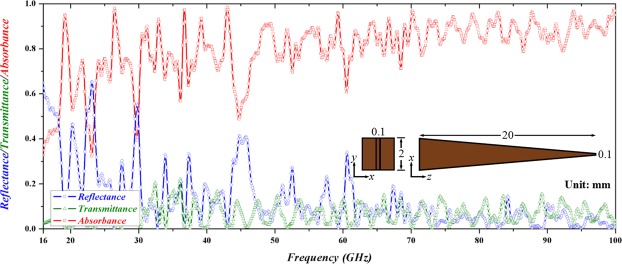
Reflectance (blue), transmittance (olive) and absorbance (red) of the all-dielectric taper. Inset indicates top and front views of the taper and its dimensions.

In FITD simulation, the boundaries in x- and y-directions are set as perfect magnetic conductor and open conditions, respectively, with an incident pulse propagating along z-direction at frequency range from 16 to 100 GHz. The calculated reflectance, transmittance and absorbance are shown in Fig. 2; the transmittance is approximately below 0.2 and the reflectance varies between 0 to 0.5 and the absorbance is larger than half of the incident power within almost the entire frequency range from 18 to 100 GHz, indicating a successful demonstration of broadband light trapping or ‘trapped rainbow’ by the proposed all-dielectric taper. In addition to the abovementioned broadband light trapping ability, the single all-dielectric taper also efficiently suppresses reflectance to trap much more power from an incident pulse by gradually altering its impedance of the structure in accordance with effective medium theory^28^.

### Power flow distribution and energy vortices

**Figure 3:**
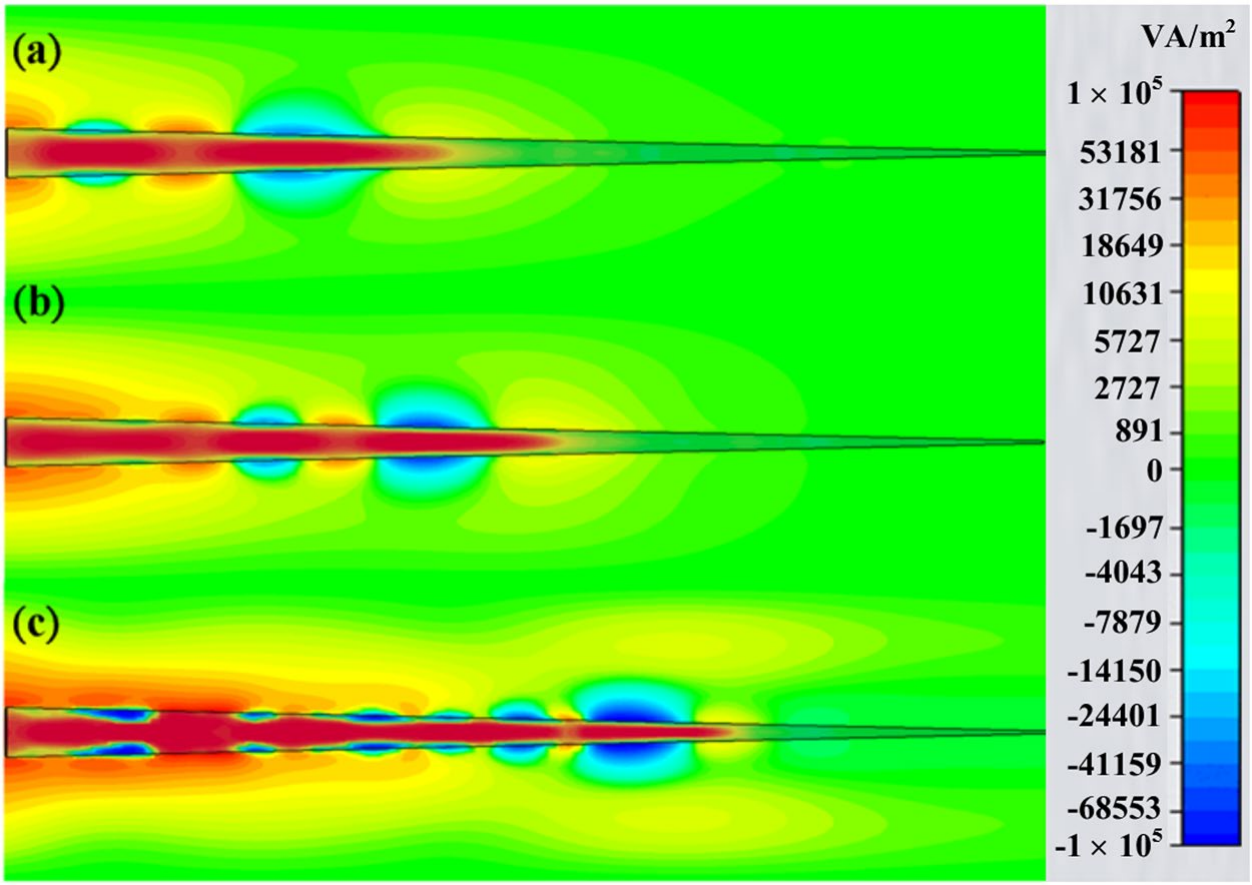
Top view of the power flow distributions of the all-dielectric taper at the frequency of 23.486 GHz (**a**), 27.528 GHz (**b**) and 36.641 GHz (**c**), respectively. Opposite propagation directions of the power flow would cancel out the propagation distances of an incident wave and result in zero propagation distance and effective zero group velocity. The critical thicknesses for the three frequencies are approximately 14.4 mm for (**a**), 1.2 mm for (**b**) and 0.84 mm for (**c**), respectively.

To realize the hidden mechanism of such high absorbance achieved by the all-dielectric taper, we monitor power flow distributions in FITD simulation at three different frequencies of 23.486, 27.528, and 36.641 GHz, respectively, as portrayed in Fig. 3. Note that the frequencies are chosen based on minimum reflectance instead of maximum absorbance in order to dilute influences from reflectance. Within these three different frequencies, there indeed appear positive power flows and negative power flows within and adjacent to the taper, respectively. These opposite signs of power flows formed energy vortices. The developed energy vortices eventually trap incident waves and engender zero propagation distances and zero group velocities, the utmost goal of slow light systems. In simulation, the light is trapped at different thicknesses of about 1.44, 1.2, and 0.84 mm for the frequency components of 23.486, 27.528, and 36.641 GHz, respectively. Besides, it is worth mentioning that even most incident power is trapped within the all-dielectric taper by exciting the corresponding surface modes, there are still some leakage waves, suggested by non-zero transmittance. The potential causes are low quality factor of the taper, and non-zero reflectance from imperfectly matched impedance with free space.

### Lewin’s theory and retrieved constitutive parameters

If we further scrutinize into the power flow distributions of the taper, we could observe that such excitation of the surface modes behaves very similar to the excitation of negative Goos-Hänchen effect in an NRW; both possess a positive power flow within the core and negative power flows in the claddings. In fact, we can relate electric and magnetic scattering coefficients to effective constitutive parameters of the scattering object under small particle approximation (i.e., only the first electric and magnetic modes are excited) in the aids of Lewin’s theory. Thus, we can disclose the relationship between surface modes and negative Goos-Hänchen effect in negative refractive waveguide. Lewin’s theory, which is widely employed to explain novel phenomena of dielectric metamaterials^29,30^, used Stratton’s solution of scattered electric field by a sphere to match the boundary conditions at the interface of the sphere and environment. Then, the relation between scattering coefficients and effective constitutive parameters is derived as shown below^31^

2-1 $${a}_{1}=-i\frac{2}{3}{({k}^{2}{\varepsilon }_{m}{\mu }_{m})}^{\frac{3}{2}}{a}^{3}\frac{{\mu }_{m}-{\mu }_{eff}}{2{\mu }_{m}+{\mu }_{eff}}$$

**Figure 4:**
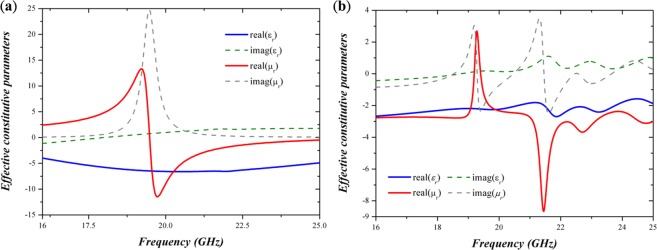
Retrieved constitutive parameters of (**a**) the shorter and (**b**) longer dielectric tapers. From these figures, we could achieve negative indices of −6 and −2, respectively. Notice that, the retrieved parameters of the longer taper are not robust due to violation of small-particle approximation; yet, we can still obtain an insight of the taper, for example, the retrieved constitutive parameters of the taper suggest much higher reflectance at the frequencies around 19 and 21.5 GHz due to the opposite signs and distinguished values of permittivity and permeability, respectively, which is consistent with the simulation.

2-2 $${b}_{1}=-i\frac{2}{3}{({k}^{2}{\varepsilon }_{m}{\mu }_{m})}^{\frac{3}{2}}{a}^{3}\frac{{\varepsilon }_{m}-{\varepsilon }_{eff}}{2{\varepsilon }_{m}+{\varepsilon }_{eff}}$$

where *k* indicates free space *k*-vector, *ε*_*m*_ and *μ*_*m*_ the constitutive parameters of the environment (i.e., air in our case), and ‘*a*’ an effective radius of the scattering object. Figure 4 reveals the corresponding effective constitutive parameters derived from the calculated scattering coefficients for both the shorter and longer dielectric taper. Clearly, there exists a band of negative refraction with a maximum value of around −6 for the shorter taper as portrayed in Fig. 4(a); as for the longer taper, Fig. 4(b) shows a negative refractive band within almost entire simulated region with a smaller index of around −2 to −3. Note that although we cannot use Lewin’s theory to calculate robust effective constitutive parameters of the taper due to violation of small particle approximation (*a*_2_ and *b*_2_ are not approaching to zero for the taper), yet we can still get an insight of broadband negative index if only considering the first electric and magnetic scattering coefficients. For example, there appear opposites signs and distinguished values of permittivity and permeability at the ranges from 19 to 20 and 21 to 22 GHz, respectively, which could be potential sources of large reflectance among these frequencies as suggested in Fig. 2. In a short summary, we could affirm that the excitation of the surface modes of the taper could be analogue to activation of negative Goos-Hänchen by the all-dielectric taper with negative refractive index and the high absorbance of the taper is attributed to the formation of an energy vortex, also a character of negative Goos-Hänchen effect.

**Figure 5:**
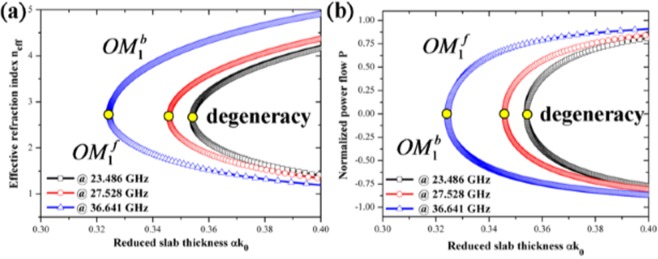
Schematic diagram for the variation of effective refraction index (**a**) and normalized power flow (**b**) with the reduced slab thickness at three different frequencies. The refractive indices of the core materials at three different frequencies are −6.04, −6.16 and −6.5, respectively. The cladding materials are air for the three frequencies. From (**a**), the effective refraction index of the NRW, smaller than the one of the components of the all dielectric taper suggests the mechanism of the slowing light effect stemming from the negative Goos-Hänchen effect instead of large group refraction index. From (**b**), the reduced slab thicknesses where the effective group velocities are zero at the three frequencies are 0.363, 0.357, and 0.329, respectively.

Since the similarity between the surface modes of the all-dielectric taper and oscillatory modes of an NRW and indeed we could retrieve a negative index for the taper in the aids of Lewin’s theory, we might analyze the taper by the waveguide theory proposed in refs^10,11^. In calculation, we utilize refractive indices of the core materials equal to −6.04, −6.16 and −6.5, respectively for the three different frequencies and the two cladding materials in the all three cases are air with ε_r_ and μ_r_ equal to 1. Note that the refractive indices are chosen to match the critical thicknesses provided by the theory and the one obtained in FITD. The first oscillatory modes at the three different frequencies are illustrated in Fig. 5 for the diagrams of the variation of the effective refraction index and the normalized power flow ${\rm{P}}=\frac{{P}_{1}+{P}_{2}+{P}_{3}}{|{P}_{1}|+{P}_{2}|+|{P}_{3}|}$ with the reduced slab thickness αk_0_ where P_1_, P_2_, and P_3_ are power flow at the upper cladding, core, and lower cladding, respectively. The fundamental degeneracy oscillatory points correspond to the reduced slab thickness αk_0_ = 0.363, 0.357, and 0.329, respectively. It is worth noting that the effective index of the system is smaller than each component of the taper (i.e., zirconia and air) at the degeneracy points, suggesting that the mechanism of slow light stems from negative Goos-Hänchen effect instead of a large group refraction index. Also, since the reduced slab thicknesses of the first degeneracy mode is around 0.37 to 0.33 for different frequencies and the maximum and the minimum supported critical thicknesses of the all-dielectric taper should be 2 and 0.1 mm, respectively in our design, we can therefore predict that the lower and upper limits of the operating frequency are thus about 17.65 and 330 GHz, respectively that agree the ones obtained in the FITD simulation results (not shown here) where the transmittance drastically increases beyond the frequency of 330 GHz, suggesting that the negative Goos-Hänchen effect is no longer supported by the all-dielectric taper beyond this upper frequency limit.

### Finite-element based simulation and experimental measurement

**Figure 6:**
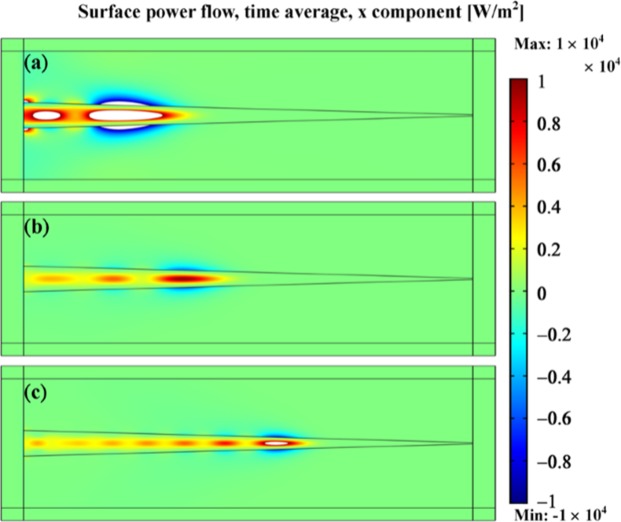
Snapshots of power flow distributions for frequencies at 23.486 GHz (**a**), 27.528 GHz (**b**), and 36.641 GHz (**c**). Refractive indices utilized here are identical to the ones obtained in the analytical solution and the critical thicknesses at (**a**), (**b**) and (**c**) are 1.48, 1.24, and 0.86 mm, respectively. Thus, we could affirm that the calculated refraction indices from FITD simulation and the analytical results work very well in finite-element simulation with very consistent critical thicknesses.

**Figure 7:**
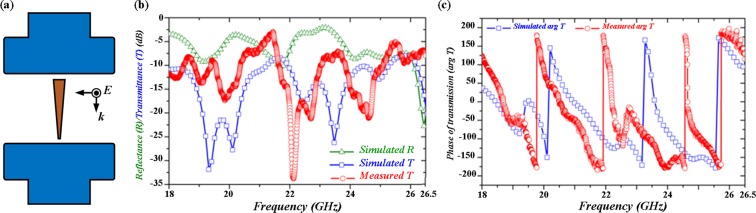
(**a**) Scheme of experimental measurement setup. The dielectric taper is sandwiched between WR-42 waveguides connected to vector network analyzer through cable lines. (**b**) Simulated transmittance (blue) and measured transmittance (red) curves in dB scale at a frequency range spanning from 18.0 to 26.5 GHz. Multi transmittance dips are observed in the spectra which suggest the potential frequencies where the negative Goos-Hänchen effect occurs. (**c**) Transmittance phase change in simulation (blue) and measurement (red). Both measured magnitude and phase are similar to the simulated results with a frequency offset of less than 1.5%.

To validate the refractive indices retrieved from the theory and numerical simulation, we employ finite-element-based (FEM) electromagnetic solver to further verify their behaviors. Here, the dimensions of the all-dielectric taper with the retrieved indices are used in FEM; squares surrounded the taper are perfect matched layers to absorbs the scattered field in the FEM simulation. As shown in Fig. 6(a–c), the positive and negative power flows within and adjacent to the taper can be clearly observed, indicating occurrence of negative Goos-Hänchen effect. The different frequency components of an incident pulse with wavelengths of 12.77, 10.90, and 8.19 mm stop at the critical thicknesses of 1.48, 1.24, and 0.86 mm, respectively in FEM simulation, which possesses only an offset of 3.2% in terms of *αk*_0_ compared to the results obtained in FITD simulation. Hence, the retrieved effective index works well in FEM based simulation and provides consistent results. Finally, to experimentally demonstrate broadband light trapping, the designed all dielectric taper was bought from MeiTek Inc. with a length tolerance of 0.1 mm and characterized by a vector network analyzer (VNA, Agilent E8364A). Standard waveguides (WR-42) are attached to VNA and emitted electromagnetic waves with transverse electrical mode (TE_10_) within a frequency range from 18.0 to 26.5 GHz. Then we measure both the magnitude and phase of transmittance, respectively. To mimic the boundary condition of perfect magnetic conductor, we periodically arrange the taper along x-direction. Also, due to the lack of the waveguides at other frequency ranges, we focus our measurement within K band instead of the entire frequency range portrayed in simulation. As shown in Fig. 7(a,b), the measured magnitude of transmittance follows similar trend compared to the simulated results with several transmission dips with a frequency offset of less than 1.5%. The measured phase of transmittance is also similar to the simulated results. The deviation may stem from the different dielectric constant in experiments and in simulation. The fabrication tolerance could be another possible reason.

## Discussion

In this work, we excite the surface modes of the all-dielectric taper that is composed of the monolithic dielectric material, ZrO2 with dielectric constant *ε*_*r*_ = 33 and loss tangent tan*δ* = 0.02 to successfully achieve broadband light trapping phenomena or so-called ‘trapped rainbow’ both in simulation and in the experimental measurement. The power flow distributions at three different frequencies are then recorded to demonstrate positive and negative power flows within the core and adjacent to the claddings, respectively at different critical thicknesses. Such excitation of the surface modes could be analogue to negative Goos-Hänchen effect predicted by negative waveguide theory with the retrieved broadband negative index around −6. Thus, the function of metal to create single or double negative identities is comprehensively replaced by the all-dielectric taper based on Mie resonance and Lewin’s theory and such employment not only decreases Ohmic losses from metal but also maintains slow light effect and increases photons-trapping time as much as possible. Finally, we provide analysis of the taper based on negative waveguide theory and further verify the obtained parameters by FEM simulation. These simulations as well as measurement results agree each other very well.

## Method

### Calculation of scattering coefficients in simulation

Due to the lack of an exact solution of scattering coefficient for an arbitrarily shaped object, we employ finite element simulation to extract out the corresponding parameters. First, we embed the dielectric taper into a spherical shell. The shell is set as perfect matched layer in simulation to absorb outgoing waves. Then, the corresponding scattering coefficients could be obtained by recording the total electric field (i.e., incident field + scattering field) at the inner boundary of the spherical shell. The scattering coefficient is obtained through orthogonality of spherical elementary waves formulated as shown below

M-1 $${a}_{n}=\frac{{\int }_{0}^{2\pi }\,{\int }_{0}^{\pi }\,{E}_{total}\cdot {M}_{o1n}sin\theta d\theta d\phi }{{\int }_{0}^{2\pi }{\int }_{0}^{\pi }{M}_{o1n}\cdot {M}_{o1n}sin\theta d\theta d\phi }$$

M-2 $${b}_{n}=\frac{{\int }_{0}^{2\pi }\,{\int }_{0}^{\pi }\,{E}_{total}\cdot {N}_{e1n}sin\theta d\theta d\phi }{{\int }_{0}^{2\pi }\,{\int }_{0}^{\pi }\,{N}_{e1n}\cdot {N}_{e1n}sin\theta d\theta d\phi }$$

where *E*_*total*_ is the recorded field at the boundary and *M*_*o1n*_ and *N*_*e1n*_ are vector spherical wave functions^26^.
